# Editorial: Quantitative Analysis of Neuroanatomy

**DOI:** 10.3389/fnana.2015.00143

**Published:** 2015-11-11

**Authors:** Julian M. L. Budd, Hermann Cuntz, Stephen J. Eglen, Patrik Krieger

**Affiliations:** ^1^Department of Informatics, School of Engineering and Informatics, University of SussexBrighton, UK; ^2^Ernst Strüngmann Institute for Neuroscience in Cooperation with Max Planck SocietyFrankfurt/Main, Germany; ^3^Frankfurt Institute for Advanced StudiesFrankfurt/Main, Germany; ^4^Department of Applied Mathematics and Theoretical Physics, Cambridge Computational Biology Institute, University of CambridgeCambridge, UK; ^5^Department of Systems Neuroscience, Medical Faculty, Ruhr University BochumBochum, Germany

**Keywords:** neuroinformatics, connectome, mathematical modeling, statistical analysis, quantitative methods, computational neuroscience

## Introduction

The true revolution in the age of digital neuroanatomy is the ability to extensively quantify anatomical structures and thus investigate structure-function relationships in great detail. Large-scale projects were recently launched with the aim of providing infrastructure for brain simulations. These projects will increase the need for a precise understanding of brain structure, e.g., through statistical analysis and models.

From articles in this Research Topic, we identify three main themes that clearly illustrate how new quantitative approaches are helping advance our understanding of neural structure and function. First, new approaches to reconstruct neurons and circuits from empirical data are aiding neuroanatomical mapping. Second, methods are introduced to improve understanding of the underlying principles of organization. Third, by combining existing knowledge from lower levels of organization, models can be used to make testable predictions about a higher-level organization where knowledge is absent or poor. This latter approach is useful for examining statistical properties of specific network connectivity when current experimental methods have not yet been able to fully reconstruct whole circuits of more than a few hundred neurons.

## Reconstruction

The first theme illustrates how novel quantitative anatomical methods are reducing the time and effort taken to reconstruct neurons and networks even when data are incomplete.

Modeling the electrophysiological computations made by single neurons requires a precise reconstruction of their morphology. Blackman et al. (2014) assessed the accuracy of neuronal reconstructions of biocytin-labeled cells against reconstructions from fluorescence-based imaging using 2-photon microscopy. The authors conclude that biocytin-labeled cells are more accurate at reproducing diameter values which is in particular crucial for electrophysiology modeling, while faster fluorescence-imaging reconstruction methods are appropriate for tasks such as cell-type classification.

Identifying cell types based on accurate tracings is very time-consuming. From such tracings of microscopic images, it is known that retinal cell types can be identified by their laminar position in the network (Sümbül et al., 2014a). Taking advantage of this link between macroscopic and microscopic features, Sümbül et al. (2014b) show how automated volumetric reconstructions can be performed more rapidly using the fluorescence distribution directly obtained from the image stacks.

Integrating structural and functional data has always been central to reconstructing neural circuits. Here, Ullo et al. (2014) apply a novel method that uses structural data of *in vitro* neuronal networks to constrain estimates of functional connections underlying spiking data of the same network acquired with microelectrode arrays. The general idea could also apply to the more complex structures *in vivo*.

Our understanding of synaptic connectivity is largely based on measurements from brain slice preparations. However, some of the complex 3D geometry of neurons is unavoidably lost by slicing. This problem affects connectivity measurements, especially for long-range connections. Two articles address this problem. First, van Pelt et al. (2014) validate a statistical approach (implemented in the NETMORPH software) for inferring complete neuronal reconstructions from incomplete slice data. From these completed neuronal morphologies, the authors explain how this information can be used to estimate connectivity in large-scale networks. Second, Miner and Triesch (2014) use a computational model to propose how differences in the experimental procedure such as slice thickness and sampling area can explain differences observed in experimentally-derived results.

Abnormalities in subcellular organelle morphology and distribution characterize a variety of neuropathological conditions. To aid faster quantification, Perez et al. (2014) combine image processing methods with a supervised, multi-resolution machine learning algorithm to automatically segment specific types of cellular organelles (mitochondria, lysosomes, nuclei, and nucleoli) from electron microscopy (EM) image stacks. The authors demonstrate how this approach should generalize to other organelle types and scale to large 3D datasets from serial electron microscopy.

## Description

The second theme in this Research Topic is that mathematical techniques are applied to describe the spatial properties of neurons and networks at a range of scales (Eglen et al., 2008; Hansson et al., 2013). Firstly, two papers used spatial statistics to examine spatial patterning within a region of neural tissue. Anton-Sanchez et al. (2014) studied the spatial distribution of synapses in layers I to VI of rat cortex in three dimensions. They found that synapses are distributed randomly, subject only to not physically overlapping with each other, although density variations were found between different layers. Moving from the distribution of synapses to distributions of neurons, Keeley and Reese (2014) proposed a new metric for evaluating the spatial regularity in two-dimensional distributions of neuronal somata. They suggest a normalization term for the widely-used regularity index measure that compares the average and the standard deviation of the distance between cells. Using various genetically inbred strains their new measure is found to be effective in detecting genes controlling spatial patterning in various genetically inbred strains using quantitative trait loci approaches.

The papers by Anton-Sanchez et al. (2014) and Keeley and Reese (2014) both analyse the distribution of synapses and neurons by treating these objects as points in space. Polavaram et al. (2014) by contrast studied the detailed morphology of individual neurons to search for key features underlying the variability in axonal and dendritic morphologies. By comparing around 5000 neuron morphologies curated from the Neuromorpho.org repository, they discovered six main morphological classes, with clustering driven mainly by biological factors, such as cell type, rather than technical factors, such as recording laboratory.

The study by Polavaram et al. (2014) highlights the difficulties in performing quantitative analysis of data recorded across many laboratories. Instead of reanalysing raw data, Beul and Hilgetag (2014) performed a detailed literature review of rodent cortex anatomy to evaluate the evidence for a universal “canonical microcircuit” (Douglas and Martin, 2004). Beul and Hilgetag suggest such a canonical microcircuit, proposed based on data from cat striate cortex, is unlikely to apply into other regions of the cortex where the granular layer (layer 4) is reduced or absent—the “agranular areas” of cortex. Instead, they propose a revised wiring diagram, with reduced inhibition between upper and deep layers in these agranular regions.

## Generative modeling

The third and final theme that emerged from this collection of articles concerns the usage of generative methods to bridge the gap between single neurons and the overall network structure (Budd and Kisvárday, 2012). Computational models sometimes based on simple self-organizing principles exist that reproduce biology at a high level of detail both at the microscopic and macroscopic scales (Schneider et al., 2014). Complementing these approaches, large databases have emerged that embed the biological details captured at the microscopic scale into the context of larger scale structures (Chiang et al., 2011). The resulting generative approaches allow for a better intuition of the underlying principles for higher-level organization and for making predictions in cases where data are currently sparse or missing.

Egger and colleagues provide a software package *NeuroNet* that generates a statistical connectome model of the barrel cortex while taking into account statistical measures of synapse and soma distributions as well as a small subset of complete realistic cellular morphologies for all cell types (Egger et al., 2014). The resulting statistical connectome is in line at all scales with experimental anatomical and electrophysiological measurements in barrel cortex and indicates that cortical connections are probabilistic as a function of dendrite and axon overlap.

In the study by Egger et al. registering the reconstructed morphologies and synaptic positions to the barrel outlines is essential to generate their statistical connectome. In a similar line, the context in the circuit is the defining feature of the study by Torben-Nielsen and de Schutter that provides a software tool in Python called *NeuroMaC* to generate synthetic dendritic morphologies within the constraints provided by a given piece of tissue (Torben-Nielsen and De Schutter, 2014). The synthetic neuronal morphologies are grown both considering the context of large-scale circuit features and the interactions with other growing dendritic trees in the vicinity.

Aćimović and colleagues study the relationship between dendritic shape and network measures using statistical models at both micro- and macroscopic scales albeit in a simplified setting that allows analytical solutions to be obtained (Acimovic et al., 2015). For analytical tractability, the small scale dendritic and axonal shapes are described by density fields that predict network motif distributions and can be compared with experimental data (Song et al., 2005; Rieubland et al., 2014).

Brain disorders are often accompanied by alterations in the higher level context in which neurons are embedded. Using a model of structural plasticity, Butz et al. (2014) analyse how such global structural changes can affect network connectivity. It is argued that local homeostatic structural plasticity mechanisms can cause changes in network topology.

With the parametric anatomical modeling (PAM) technique by Pyka et al. (2014) the precise shape of macroscale anatomical structures is incorporated into neural network models. Using empirically-based mapping rules and connectivity kernels to generate realistic pathway trajectories, spatial connectivity matrices, and axonal conduction distances, Pyka and colleagues make testable predictions for interlaminar connectivity parameter distributions.

Finally, long-range connections require fast and reliable axonal signal propagation and Neishabouri and Faisal (2014) have studied a recently observed structural formation of proteins and lipids known as lipid rafts (Pristerà et al., 2012) in thin, unmyelinated axons in the peripheral nervous system. Using realistic stochastic modeling of individual ion channels, Neishabouri and Faisal show that while action potential conduction in such systems was reliable, it did not offer any obvious gain in either conduction velocity or metabolic cost over a uniform ion channel density.

## Conclusion

With these articles we hope the reader will appreciate that understanding neural structure quantitatively and its functional relations is more than a handle turning exercise of known algorithms but a creative interdisciplinary endeavor of a variety of approaches across different species, brain regions, and spatial scales. Here, authors have managed to coax information out of noisy data obtained at the extremes of methodological resolution; they have discovered new ways of describing anatomical organization; and arrived at novel ideas that when implemented in a generative way provide an anatomical framework for large-scale network models of the brain. To maximize this creativity from limited funding, there is an obvious need to provide an environment in which individual exploration, data access, collaboration, and reproducibility is made much easier through an open and shared informatics framework (Green et al., 2015).

### Conflict of interest statement

The authors declare that the research was conducted in the absence of any commercial or financial relationships that could be construed as a potential conflict of interest.
